# Comprehensible Machine-Learning-Based Models for the Pre-Emptive Diagnosis of Multiple Sclerosis Using Clinical Data: A Retrospective Study in the Eastern Province of Saudi Arabia

**DOI:** 10.3390/ijerph20054261

**Published:** 2023-02-27

**Authors:** Sunday O. Olatunji, Nawal Alsheikh, Lujain Alnajrani, Alhatoon Alanazy, Meshael Almusairii, Salam Alshammasi, Aisha Alansari, Rim Zaghdoud, Alaa Alahmadi, Mohammed Imran Basheer Ahmed, Mohammed Salih Ahmed, Jamal Alhiyafi

**Affiliations:** 1 College of Computer Science and Information Technology, Imam Abdulrahman Bin Faisal University, P.O. Box 1982, Dammam 31441, Saudi Arabia; 2 Department of Computer Science, Kettering University, Flint, MI 48504, USA

**Keywords:** pre-emptive diagnosis, multiple sclerosis, machine learning, explainable artificial intelligence, shapley additive explanation, local interpretable model-agnostic explanations

## Abstract

Multiple Sclerosis (MS) is characterized by chronic deterioration of the nervous system, mainly the brain and the spinal cord. An individual with MS develops the condition when the immune system begins attacking nerve fibers and the myelin sheathing that covers them, affecting the communication between the brain and the rest of the body and eventually causing permanent damage to the nerve. Patients with MS (pwMS) might experience different symptoms depending on which nerve was damaged and how much damage it has sustained. Currently, there is no cure for MS; however, there are clinical guidelines that help control the disease and its accompanying symptoms. Additionally, no specific laboratory biomarker can precisely identify the presence of MS, leaving specialists with a differential diagnosis that relies on ruling out other possible diseases with similar symptoms. Since the emergence of Machine Learning (ML) in the healthcare industry, it has become an effective tool for uncovering hidden patterns that aid in diagnosing several ailments. Several studies have been conducted to diagnose MS using ML and Deep Learning (DL) models trained using MRI images, achieving promising results. However, complex and expensive diagnostic tools are needed to collect and examine imaging data. Thus, the intention of this study is to implement a cost-effective, clinical data-driven model that is capable of diagnosing pwMS. The dataset was obtained from King Fahad Specialty Hospital (KFSH) in Dammam, Saudi Arabia. Several ML algorithms were compared, namely Support Vector Machine (SVM), Decision Tree (DT), Logistic Regression (LR), Random Forest (RF), Extreme Gradient Boosting (XGBoost), Adaptive Boosting (AdaBoost), and Extra Trees (ET). The results indicated that the ET model outpaced the rest with an accuracy of 94.74%, recall of 97.26%, and precision of 94.67%.

## 1. Introduction

Chronic diseases are generally identified as illnesses that tend to last over a long period, requiring continuing medical attention and causing limitations and disabilities [1]. According to the World Health Organization (WHO), 41 million people die of chronic diseases yearly [1]. Chronic diseases are not just inherited but are also caused by exposures throughout life [2]. Many chronic diseases have been related to lifestyle habits, such as smoking, consuming unhealthy foods, and not being physically active [3]. Even though an early diagnosis is crucial in managing chronic diseases, they often exhibit no symptoms in their early stages, necessitating the emergence of the latest technologies contributing to the pre-emptive diagnosis of these diseases.

Several chronic diseases, including Multiple Sclerosis (MS), are prevalent in Saudi Arabia. MS affects approximately 2.8 million individuals worldwide. Since 2013, the prevalence of MS has increased [4]. According to Al Jumah et al. [5], the prevalence of MS in Saudi Arabia was greater in the central region and lower in the southern region. Moreover, 40.40 per 100,000 of all populations and 61.95 per 100,000 Saudi citizens were diagnosed with MS. Females were also shown to be more likely than males to develop MS at a ratio of 2:1, and young, educated individuals are more likely to be affected in various Saudi Arabian regions [5]. 

MS is a lifelong chronic inflammatory demyelination illness that can damage the spinal cord (central nervous system) and the brain, causing the immune system to attack the myelin that protects nerve fibers. MS causes miscommunication between the brain and the rest of the body, leading to major disability [6]. Many potential symptoms that vary from one patient with MS (pwMS) to another are experienced, such as cognitive deficiencies, weakness, sensory impairment, visual loss, dizziness, and spasticity [7]. Since MS affects everyone differently, there is presently no reliable approach to anticipate how the condition will progress in a particular pwMS. In addition, some pwMS may appear healthy for years after the diagnosis, while others may advance more swiftly. Furthermore, it is currently not known whether MS can be cured. However, it has been found that disease-modifying medications help to manage symptoms and stop the course of the disease [8]. Therefore, screening for MS before symptoms develop and following early treatment plans are crucial to improving the patient’s quality of life [9]. 

As disease-modifying medications help in the symptomatic treatment and disease progression, an accurate and reliable MS diagnosis is essential for enabling pre-emptive therapies for the disease [10]. In addition to ruling out any other conditions that might resemble MS clinically or radiologically, MS is diagnosed by having central nervous system lesions that are distinct from one another in both time and space [11]. For the disease’s diagnosis, there is no specific laboratory test that can precisely identify the disease. Considering this, the most recent McDonald diagnostic criteria for MS, released in 2017, encompass clinical assessment, imaging, and laboratory data [12]. Nowadays, Magnetic Resonance Imaging (MRI) is the most effective technique for diagnosing MS, as well as tracking the disease’s progression and testing treatment effectiveness. However, utilizing MRI to diagnose MS is expensive, time-consuming, and prone to human errors [13]. 

Machine Learning (ML) is a branch of computer science that focuses on the theory of pattern recognition and computational learning. The implementation of algorithms takes place through the process of learning from data and making predictions using unseen data. The growing capabilities of ML facilitated the process of identifying patterns not visible to humans using the massive medical data available. Therefore, several studies were conducted to diagnose MS using ML and DL algorithms. However, most studies focused on diagnosing MS using imaging datasets, and only some used clinical data, which added extra workload associated with data collection and the challenge of using complexly constructed models. Therefore, by using the latest technologies, this study aims to overcome the limitations of previous work by utilizing simple clinical data to detect pwMS pre-emptively.

This study’s dataset was obtained from King Fahad Specialist Hospital (KFSH) in Dammam, Saudi Arabia. It contains clinical data records of 569 patients (365 pwMS and 205 without MS). Various ML algorithms were utilized, including Support Vector Machine (SVM), Decision Tree (DT), Logistic Regression (LR), Random Forest (RF), Extreme Gradient Boosting (XGBoost), Adaptive Boosting (AdaBoost), and Extra Tree (ET). The results showed that ET attained the highest accuracy of 94.74% with 11 features only. To better understand how an AI model reaches decisions, researchers have developed Explainable Artificial Intelligence (XAI). In this approach, ML models are modified to generate explainable models, enabling the end users to confidently manage, comprehend, and trust emerging AI systems [14]. Shapley Additive Explanation (SHAP) and Local Interpretable Model-Agnostic Explanations (LIME) were used in this study to explain the outperforming model’s findings.

This paper is divided into the following sections. Section 2 comprises a thorough literature review. Section 3 details the materials and methods used, including the dataset description, statistical analysis, a description of the employed ML algorithms, the performance measures used to assess the developed models, and finally, the optimization strategy chosen. The findings and the feature selection method utilized are also explained in Section 4, and the results of the models employing XAI approaches are described in Section 5. Finally, the conclusions and future work are discussed in Section 6.

### Study Objectives

Aiding medical personnel in pre-emptively screening and treating MS can help slow the disease’s progression. This study aimed to develop a valuable tool for predicting MS that can be deployed in local hospitals. The following is a synopsis of the study’s contributions:

- Developed the first clinically applicable and cost-effective ML model to screen MS pre-emptively in Saudi Arabia.
- Utilized the SelectKBest technique based on the chi-squared test to reduce the number of features needed to produce accurate results.
- Compared and evaluated the diagnostic performance of simple and ensemble classifiers.
- Applied Explainable Artificial Intelligence (XAI) techniques to assist medical professionals in comprehending how features affect the top-performing ML model in this study.

## 2. Literature Review

Based on clinical information and Retinal Nerve Fiber Layer (RNFL) thickness determined by Optical Coherence Tomography (OCT), a study [15] was conducted to diagnose MS better and forecast the long-term course of impairment in pwMS. The dataset was obtained from Miguel Servet University Hospital, which includes 212 records (104 healthy individuals and 108 pwMS). The ML algorithms used were Multiple Linear Regression (MLR), SVM, DT, Naive Bayes (NB), Long Short-Term Memory (LSTM), K-Nearest Neighbors (KNN), and an Ensemble Classifier (EC). The results demonstrated that the EC attained an accuracy of 87.7%, a sensitivity of 87%, a precision of 88.7%, a specificity of 88.5%, and an Area Under the Curve (AUC) of 0.8775. As for forecasting the long-term impairment course in pwMS, LSTM achieved the highest accuracy of 81.7%, sensitivity of 81.1%, precision of 78.9%, specificity of 82.2%, and AUC of 0.8165.

Using the same techniques, a recent study [16] used RNFL thickness measured by OCT to diagnose MS. Only 102 records were obtained in this study from the hospital mentioned above (30 healthy individuals and 72 pwMS) using three different Spectralis OCT protocols to perform structural assessments of RNFL thickness. The macular RNFL was measured using the fast macular thickness protocol, whereas the peripapillary RNFL was measured using both fast RNF and fast RNFL-N thickness protocols. The fast macular thickness protocol with KNN was the best acquisition procedure for MS diagnosis, achieving an accuracy of 95.8%, sensitivity of 94.4%, precision of 97.1%, specificity of 97.2%, and an AUC of 0.958. Furthermore, DT performed best for MS prognosis with an accuracy of 91.3%, a sensitivity of 90%, a precision of 92.3%, a specificity of 92.5%, and an AUC of 0.913 for the fast macular thickness protocol, and SVM for fast RNFL-N thickness protocol with an accuracy of 91.3%, a sensitivity of 87.5%, a precision of 94.6%, a specificity of 95%, and an AUC of 0.913.

Similarly, the study [17] used the dataset mentioned above from Miguel Servet University Hospital, consisting of 260 records (180 healthy individuals and 80 pwMS). The authors in this study aimed to use different ML techniques to compare axonal loss in ganglion cells observed by means of the Swept-Source OCT (SS-OCT). Three ML classifiers were used and evaluated, including DT, Multilayer Perceptron, and SVM. The DT classifier obtained the best results, with an accuracy of 97.24% and an AUC of 0.995, using RNFL data in the macular area. Consequently, the authors concluded that SS-OCT provides excellent differentiation between healthy controls and MS patients based on measurements of RNFL thickness.

Likewise, the authors in [18] obtained 96 records (48 pwMS and 48 healthy individuals) from the same hospital to use SS-OCT to diagnose MS earlier. The proposed Feed-Forward Neural Network (FFNN) classifier achieved promising results with an accuracy of 97.9%, a sensitivity of 98%, and a specificity of 98%.

By analyzing exhaled breath, the authors of [19] aimed to diagnose MS using an electronic nose (eNose). A diagnostic test tool called eNose (Aeonose) can identify volatile organic component patterns in exhaled breath. The authors tested Aeonose’s ability to distinguish between the breath patterns of pwMS and healthy people. The dataset included 253 case controls (124 pwMS and 129 healthy individuals) who each breathed into the Aeonose for five minutes. The data from exhaled air were used to construct a predictive model using an Artificial Neural Network (ANN). With a subgroup of pwMS who had not been prescribed any medication for their MS, the authors developed a second predictive model to examine the impact of drug use. With a sensitivity of 75% and a specificity of 60%, the ANN model built using the entire dataset was able to discriminate pwMS from healthy individuals. The sensitivity and specificity of the model developed using the subgroup of pwMS not taking medication and the healthy control participants were 93% and 74%, respectively.

The authors in [20] trained a Convolutional Neural Network (CNN) using brain MRI to distinguish between MS and its imitators. The CNN model achieved an accuracy of 98.8% using a total of 268 T1 and T2 weighted brain MRI scans.

More recently, the authors in [21] used CNN to predict the progression of the disease using brain MRI. The data of 373 pwMS were collected from the Italian Neuroimaging Network Initiative (INNI) repository. CNN was used to predict clinical worsening, cognitive deterioration, or both. The results showed that the clinical and cognitive worsening achieved an accuracy of 83.3% and 67.7%, respectively. On the other hand, when the system was trained using both clinical and cognitive data, it achieved 85.7% accuracy.

Furthermore, the study [22] aimed to detect MS using MRI. The dataset contains 130 brain MRI scans (30 pwMS and 100 healthy individuals). The authors used transfer learning to train the model by using SoftMax as an activation function to classify disease development. By using CNN, the model achieved an accuracy of 98.24%, specificity of 95.45%, and sensitivity of 100%.

Furthermore, the authors in [23] presented an approach that combines CNN and the two-dimensional discrete Haar wavelet transform to identify pwMS using MRI scans. The University of Cyprus’ Laboratory of eHealth provided the dataset for this study, consisting of 58 records (38 pwMS and 20 healthy individuals). The experiments on the image data attained an accuracy of 99.05%, precision of 98.43%, and sensitivity of 99.14%.

A review of the literature on the early prediction of MS revealed that most previous studies focused on diagnosing MS using imaging datasets, whereas few used clinical data. Additionally, it has been found that relatively small datasets were explored in previous studies. Therefore, this study aims to build an ML model using simple clinical features that could predict MS accurately with the least amount of workload and computation. In addition, the work provides medical specialists with a rationale for trusting the prediction using XAI techniques. Consequently, local hospitals with low incomes gain from deploying the pre-emptive diagnosis model. 

## 3. Materials and Methods

**Figure 1:**
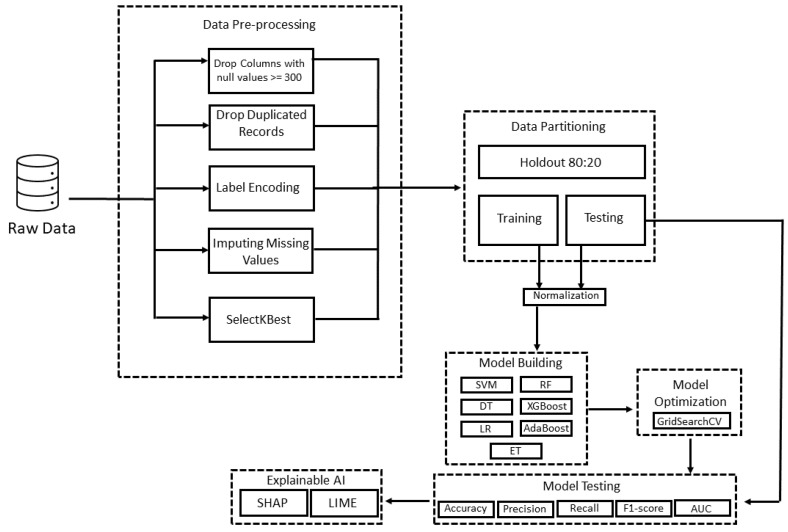
The proposed framework for the pre-emptive diagnosis of MS.

This study developed a pre-emptive model for diagnosing MS using Python programming language. A fixed seed value of 0 was set throughout all operations. Before modeling, the dataset was subjected to various pre-processing techniques, as shown in Figure 1. The SelectKBest technique with the chi-squared test and k = 11 was used to extract the best features. Additionally, the dataset was split into stratified proportions, where 80% of the data were reserved for training and were further validated using stratified 10-fold cross-validation, whereas the rest were used for testing the proposed models. The min–max scaler was then fitted to the training set and transformed into the testing set. Furthermore, seven ML algorithms were trained with the selected 11 features: SVM, DT, LR, RF, XGBoost, AdaBoost, and ET. GridSearchCV was then used with stratified 10-fold cross-validation to optimize the hyperparameters of the models using the training set. The models were assessed using a variety of performance metrics, including accuracy, precision, recall, F1-score, and AUC). Subsequently, the best model was interpreted using SHAP and LIME techniques. The process used to build the prediction models is summarized in Figure 1.

### 3.1. Data Description

The dataset used in this study was obtained from KFSH in Dammam, Saudi Arabia. The dataset includes records of 570 patients (365 pwMS and 205 healthy), with 44 demographical features and laboratory biomarkers. Table 1 demonstrates the features’ names and types. After applying the SelectKbest approach, 11 features remained, namely age, ALT (dimension), LDH, creatinine, blood urea nitrogen, total bilirubin, gamma glutamyl transferase, alkaline phosphatase, AST, platelet, and BP—systolic.

### 3.2. Statistical Analysis

**Figure 2:**
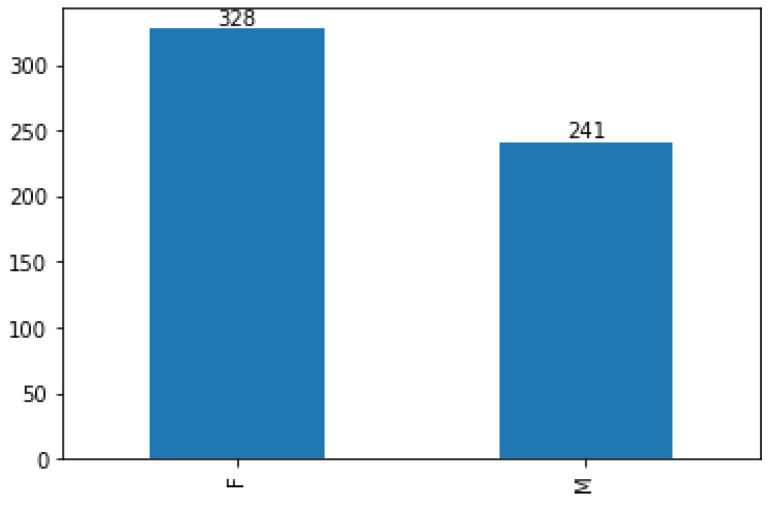
The value counts of gender.

In this section, statistical analysis was carried out to understand the data and the underlying patterns. Statistical analysis aids in determining the pre-processing methods that should be used to prepare the data for modeling. The data used in this study consisted of numerical features and only one categorical feature. The numerical attributes of the data were analyzed using well-known statistical metrics. The numerical properties of the data and their accompanying statistical breakdown are shown in Table 2. As the table demonstrated, the big difference between the 75th quartile and the maximum values indicates the presence of outliers and skewness in some features. Moreover, Figure 2 displays the value count of the gender attribute after removing the duplicates in the pre-processing stage.

### 3.3. Data Pre-Processing

One of the crucial processes in converting raw data into valuable data for training is data pre-processing. The Python Sklearn and Pandas packages were used in the current study to perform several pre-processing techniques. Initially, the dataset included 177 features and 570 records, most of which contained null values; thus, features with ≥300 null values were dropped, and any duplicated row was eliminated using the Pandas duplicate() method. Consequently, only 44 features and 569 instances remained. Furthermore, categorical data were transformed into a numerical format before training and evaluating models using the Sklearn LabelEncoder() method [24]. 

Missing values significantly influence the inferences drawn from the dataset. Therefore, it can result in several complications, including a decrease in statistical power, inaccurate parameter estimates, and difficulties with data processing. Different imputation strategies were used for missing values based on the types of attributes. In this study, the numerical null values were imputed by checking the STD to observe how the data are distributed. Whenever the STD is high, the data are more skewed; therefore, the null values are filled using the median, as shown in Equation (1), where n refers to the total number of observations.

(1) $$\mathrm{Median}=\frac{\left(n+1\right)}{2}$$

In contrast, if the STD was low, the forward-filling and the mean imputation techniques were utilized. The forward-filling method states that the nearest value before the targeted point will be utilized if the value is null. Besides the forward-filling requirement, the resampling procedure allows a maximum of one usage of each value. The subsequent missing values will be marked as missing if the closest preceding value has already been utilized once for resampling. Hence, the mean was used to impute the remaining null values, as shown in Equation (2), where *n* represents the total number of values in a column and X represents a single data point [25].

(2) $$\mathrm{Mean}\;=\frac{\sum^{\;}X}{n}$$

Next, a univariate feature selection method called Select K-Best (SelectKBest) was used. Utilizing a variety of univariate statistical tests, it chooses the K-best features from the feature set. This study selected the top 11 features using the chi-square test. Only positive features can be used in the test; hence, each non-negative and target feature receives a score from the algorithm. Pairs of expected and observed frequencies can be used to determine the score using Equation (3).

(3) $$x^{2}=\overset{n}{\underset{i=1}{\sum }}\frac{{\left(OF_{i}-EF_{i}\right)}^{2}}{EF_{i}}$$

where $OF_{i}$ is the frequency that was observed for the feature *F*’s *i*-th value, and $EF_{i}$ is the frequency anticipated for feature *F*’s *i*-th value [26].

Following pre-processing, the dataset was split into two stratified sets: 80% for training and validation and 20% for testing. The values were then scaled between 0 and 1 using the min–max scaler, which has been fitted to the training set and transformed to the testing set using Equation (4).

(4) $$\mathrm{MinMaxScaler}\;(v^{\prime }i)=\frac{x_{i}-min_{A}}{max_{A}-min_{A}}\left(new\_max_{A}-new\_min_{A}\right)+new\_min_{A}$$

where $x_{i}$ represents the *i*th value, $max_{A}$ and $min_{A}$ denote a feature’s maximum and minimum values, and $new\_max_{A}$ and $new\_min_{A}$ are the values 0 and 1, respectively. 

### 3.4. Description of Utilized Machine Learning Algorithms

#### 3.4.1. Support Vector Machine (SVM)

In 1990, Cortes and Vapnik proposed Support Vector Machine (SVM). Since then, its popularity has increased among the ML community [27]. SVM is a supervised learning algorithm that provides solutions to classification and regression problems, mainly used in binary classification problems [28]. In classification, a hyperplane is located in feature space by SVM to separate different classes [29]. The training points are mapped onto the feature space and separated by a maximum margin between classes. In the same space, the testing data points are then mapped and categorized according to which side of the margin they fall.

#### 3.4.2. Decision Tree (DT)

DTs are supervised ML classifiers that may be thought of as rule-based classifiers. Using a training set, DT develops a set of binary rules that can properly identify the majority of training set samples [30]. Thus, given a sample, DT evaluates whether multiple rules are met and produces a result. DT is straightforward and beneficial for interpretation. However, in terms of generalization, they are often not competitive with more sophisticated supervised learning algorithms and can quickly overfit if no limitations on the highest number of rules are applied [30].

#### 3.4.3. Logistic Regression (LR)

In 1958, David Cox developed LR, an ML approach based on supervised learning and statistical analysis. It is known as a log transformation of linear regression. However, unlike linear regression, it is only used for classification [31]. LR is robust and fast in predicting discrete categorical target classes [32]. Furthermore, its simplicity allows it to rapidly reach a high level of performance. Depending on the dataset, the fundamental purpose of LR is to establish linear and noncomplex decision boundaries across classes.

#### 3.4.4. Random Forest (RF)

In 2001, Leo Breiman proposed RF while introducing the concept of bagging, also known as “bootstrap aggregation” [33]. The RF classifier comprises several DTs representing various subjects from the dataset. Instead of depending exclusively on one DT, RF uses the majority vote predictions from each tree to anticipate the outcome, increasing the predictive accuracy [34].

#### 3.4.5. Extra Trees (ET)

ET is an ensemble ML classifier that Mingers first familiarized in 1989 [35]. The idea behind ET is to use several small decision trees, each of which is a weak learner on its own. ETs are comparable to other tree-based ensemble techniques, such as RF; however, unlike RFs, all the trees in an ET are trained using the same training set. Additionally, while RF simply splits a node based on variable value, ETs split a node based on both variable indexing and variable splitting values. Because of this, ETs are both generalizable and more computationally efficient than RFs [36].

#### 3.4.6. Extreme Gradient Boosting (XGBoost)

XGBoost is a model that was initially introduced by Carlos Guestrin and Tianqi Chen in 2011 and has since been continuously optimized to be used with modern data science tools and challenges [37]. XGBoost is a boosting tree-based learning framework with a high degree of expansion and versatility. It combines several models to create a robust model [37]. The most well-known benefits of XGBoost include its high scalability and parallelizability, speed of execution, and ability to frequently outperform competing algorithms. Additionally, it controls over-fitting using a more regularized model formalization, which enhances performance [38].

#### 3.4.7. Adaptive Boosting (AdaBoost)

The first genuinely effective boosting algorithm, known as Adaptive Boosting (AdaBoost), was introduced by Freund and Schapire for binary classification. It is a meta-algorithm that can enhance the performance of numerous other learning algorithms by pairing up with them [39]. AdaBoost is adaptive in the sense that cases that were incorrectly identified by earlier classifiers are considered while creating new classifiers. In other terms, the fundamental principle behind AdaBoost is to call a weak classifier repeatedly while modifying the weights given to the samples for each call [40].

### 3.5. Performance Measure

In this study, the models’ performance was assessed using a variety of performance metrics, including accuracy, precision, recall, F1-score, and AUC. In order to further assess the models, confusion matrices were used, which include True Negative (TN), True Positive (TP), False Negative (FN), and False Positive (FP), where:

- TN indicates patients who were correctly identified as non-MS patients.
- TP indicates patients who were correctly identified as pwMS.
- FN indicates patients who were incorrectly identified as non-MS patients.
- FP indicates patients who were incorrectly identified as pwMS.

Accuracy is the ratio of correctly identified MS and non-MS patients over the total number of patients in the dataset. It is mathematically represented in Equation (5).

(5) $$\mathrm{Accuracy}=\frac{\mathrm{Correctly}\;\mathrm{identified}\;\mathrm{MS}\;\mathrm{and}\;\mathrm{non}-\mathrm{MS}\;\mathrm{patients}}{\mathrm{Total}\;\mathrm{number}\;\mathrm{of}\;\mathrm{patients}\;\mathrm{in}\;\mathrm{the}\;\mathrm{dataset}}$$

Precision is the ratio of correctly identified positive instances across all predicted positive instances. It is mathematically represented in Equation (6).

(6) $$\mathrm{Precision}=\frac{\mathrm{Correctly}\;\mathrm{identified}\;\mathrm{MS}\;\mathrm{patients}\;}{\mathrm{Total}\;\mathrm{number}\;\mathrm{of}\;\mathrm{predicted}\;\mathrm{postive}\;\mathrm{instances}}$$

Recall is the ratio of correctly identified positive instances to all the positive instances in the actual class. It is mathematically represented in Equation (7).

(7) $$\mathrm{Recall}=\frac{\mathrm{Correctly}\;\mathrm{identified}\;\mathrm{MS}\;\mathrm{patients}\;}{\mathrm{All}\;\mathrm{the}\;\mathrm{positive}\;\mathrm{instances}\;\mathrm{in}\;\mathrm{the}\;\mathrm{actual}\;\mathrm{classt}}$$

Correspondingly, the F1-score is the weighted average of recall and precision. It is mathematically represented in Equation (8).

(8) $$F1-\mathrm{Score}=\frac{2\times \left(\mathrm{Precision}\times \mathrm{Recall}\right)}{\mathrm{Precision}+\mathrm{Recall}}$$

The AUC measures a classifier’s ability to distinguish between classes, as stated in Equation (9), where $n_{1}$ and $n_{0}\;$ are the numbers of negative and positive observations, respectively, and $r_{i}\;$ in $S_{0}$ = $\Sigma r_{i}\;$ denotes the degree of the *i*th positive observation.

(9) $$\mathrm{AUC}=\frac{S_{0}-n_{0}\left(n_{0}+1\right)/2}{n_{0}n_{1}}$$

### 3.6. Optimization Strategy

The hyperparameters of ML models are essential factors impacting the model performance. Setting an appropriate value for these hyperparameters can considerably enhance the model performance. The GridSearchCV technique was utilized to build models that are capable of providing accurate solutions to these problems. GridSearchCV was used in this study to find the best hyperparameters in a search space that included a range of values. It generates all possible combinations of hyperparameter values to determine the best combination using the training set. Stratified 10-fold cross-validation was used to validate the model performance. The optimal hyperparameters generated by the GridSearchCV for each algorithm are outlined in Table 3. Moreover, the hyperparameters used in the grid are outlined in the Supplementary File. 

## 4. Empirical Results

**Figure 3:**
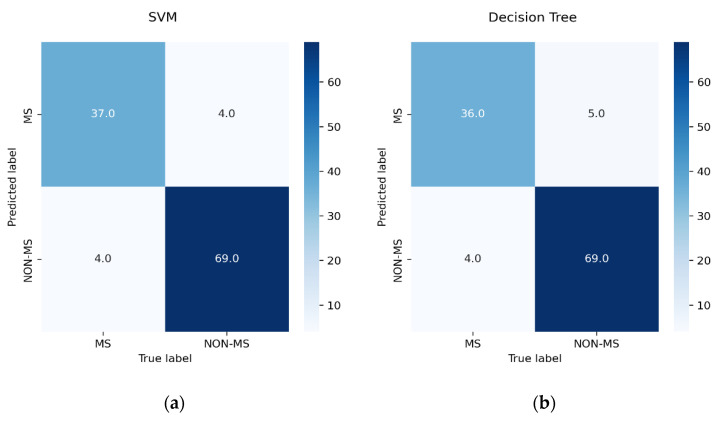

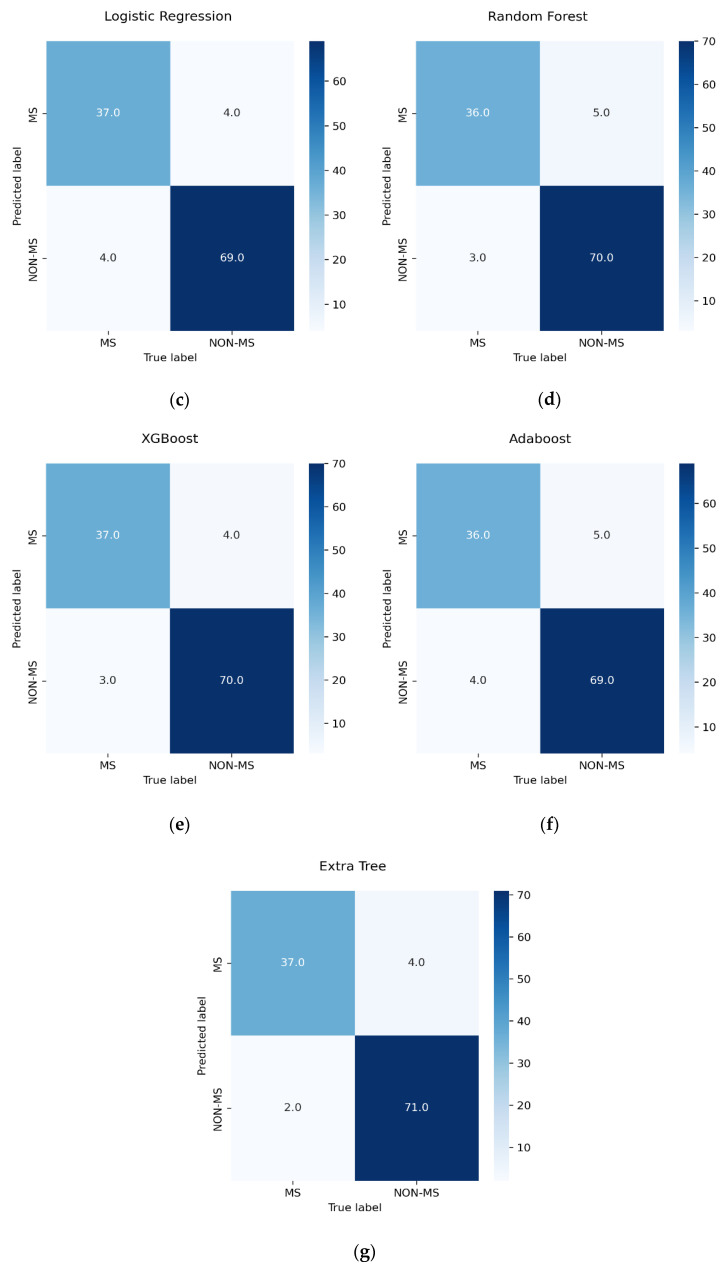
Confusion matrices: (**a**) SVM, (**b**) DT, (**c**) LR, (**d**) RF, (**e**) XGBoost, (**f**) AdaBoost, (**g**) ET.

In accordance with the described performance indicators, Table 4 assesses the developed models using the ideal hyperparameters and features subset produced by the GridSearchCV and SelectKBest techniques, respectively. Overall, the results reveal that neither overfitting nor underfitting affected the model due to the low difference between training and testing accuracy. Furthermore, the ET classifier outperformed other algorithms with an accuracy of 94.74%, a precision of 94.67%, a recall of 97.26%, and an F1-score of 95.95%. Following that, XGBoost achieved an accuracy of 93.86%, a precision of 94.59%, a recall of 95.89%, and an F1-score of 95.24%. Algorithms including SVM, LR, and RF achieved identical accuracies after implementing GridsearchCV. RF, however, performed differently in terms of precision and recall. On the other hand, DT and AdaBoost achieved the lowest performance measures after optimization with an accuracy of 92.11%, a precision of 93.24%, a recall of 94.52%, and an F1-score of 93.88%. Figure 3 displays the confusion matrices for optimized selected models.

Figure 3 reveals that the ET model, which achieved the fewest FNs (two), is the best algorithm for predicting unwanted occurrences of the targeted disease, followed by the RF and XGBoost models, which failed to classify three cases of MS. Meanwhile, ET, XGBoost, LR, and SVM achieved the lowest rates of FP. Misdiagnosis of MS may occur due to pressure to deliver a timely diagnosis, as several alternative diagnoses may mimic MS, such as functional neurologic disorders, migraines, and arterial disease [41]. The misdiagnosis of MS could lead to serious repercussions, including losing the chance for early treatment and possibly accelerating the course of the condition. Moreover, the risk of prolonged, unnecessary healthcare hazards and death is often attributed to misdiagnosed MS patients [42]. To determine the best-performing model, the lowest FN and FP values must be achieved. Therefore, it is concluded that the ET outperforms all other models for the pre-emptive diagnosis of MS.

**Figure 4:**
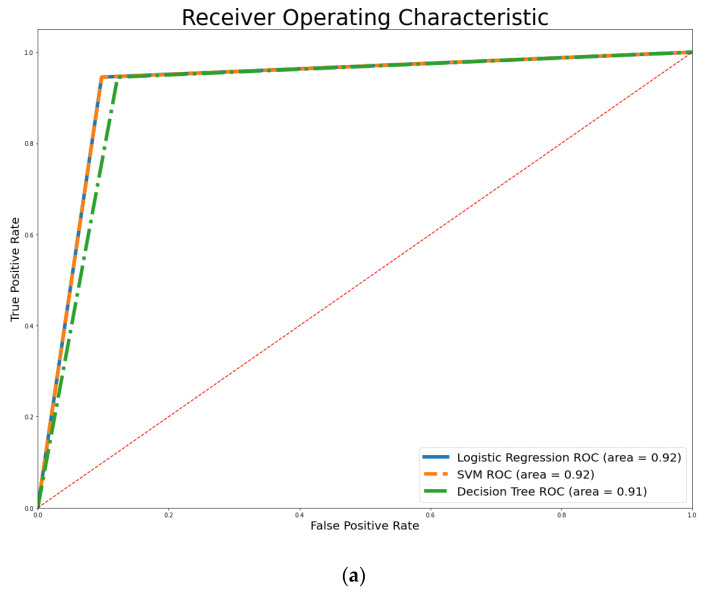

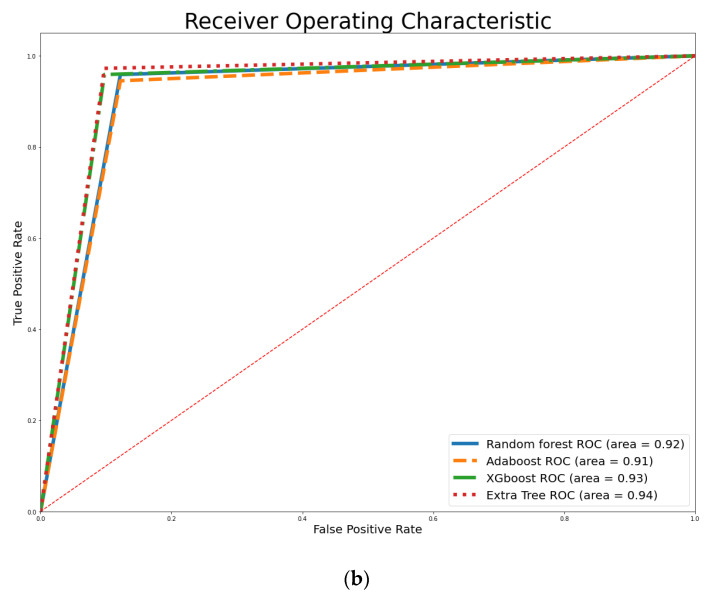
AUC values: (**a**) weak classifiers, (**b**) ensemble classifiers. (The red diagonal shows the AUC of 0.5m which separates the line to above or under 50%).

Figure 4 illustrates the Receiver Operating Characteristics (ROC) curve that evaluates the discrimination ability of the classifiers with different thresholds. Accordingly, Figure 4 reveals that the AUC values for the executed classifiers ranged from 0.91 to 0.94. However, XGBoost and ET achieved the highest values at 0.93 and 0.94, respectively. 

### 4.1. Interpretation of the Final Recommended Model

Since ML applications have become more popular over the past eight years or so, they are now having a major influence on humanity in various ways, such as lending decisions and making judgments. However, because most models are opaque by nature, mindlessly implementing their recommendations in applications that affect people could result in problems with justice, safety, and reliability, among many other concerns [43]. Accordingly, this has caused a branch of AI known as XAI to emerge, which is an essential component of enhancing the trust and dependability of AI and ML. Currently, many techniques, most notably ML and DL techniques, are not visible in how they operate and are hence referred to as black-box models. In order to gain adequate trust, and in some situations, achieve even greater performance through human–machine collaboration, XAI is particularly focused on comprehending or interpreting the judgments made by the proposed opaque or black-box models. However, it has been recognized that this poses serious issues for several industries, including health sciences and criminal justice. Consequently, arguments have been made in favor of AI that is explicable [44]. Therefore, this work implements two XAI techniques, including SHAP and LIME.

#### 4.1.1. Shapley Additive Explanation (SHAP) 

Black-box ML models are frequently used, making comprehending their results challenging. Accordingly, explainable ML algorithms that dissect the results are used to identify characteristics that influence the model’s output [45]. SHAP is one of the proposed methods, explaining each feature’s impact on the model and permitting both local and global analysis for the intended dataset. Each case prediction is proved using this method by computing all impact-considered attributes and employing SHAP values generated from the coalition game theory. The effect of each attribute on the SHAP value is roughly averaged across all possible permutations. Furthermore, the absolute SHAP value represents the degree of the feature’s impact on model prediction, making it possible to utilize it as a measurement of feature relevance [46].

**Figure 5:**
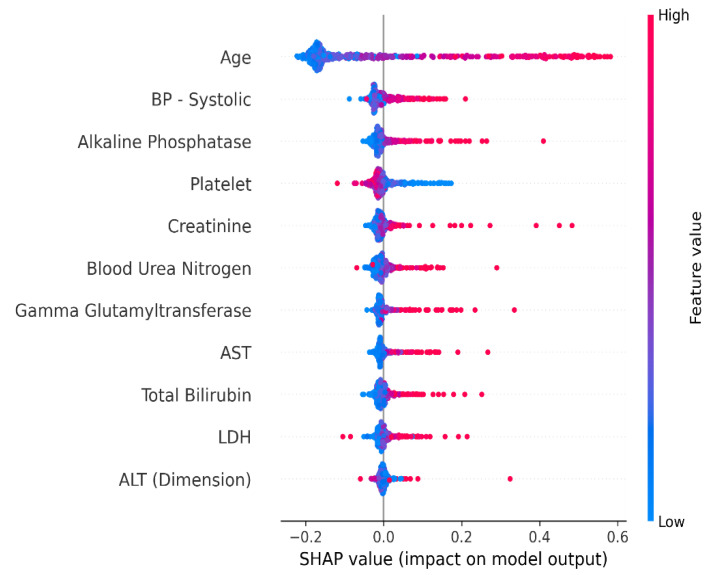
Shapely values using the ET model.

Figure 5 reveals that the high values of all features, except platelets, strongly influence the prediction, whereas their low values negatively influence the positive prediction. Overall, features including “Age”, “BP- Systolic”, and “Alkaline Phosphatase” have significant importance in model prediction, whereas those including “ALT (Dimension)” and “LDH” have a slight effect in comparison to other features.

#### 4.1.2. Local Interpretable Model-Agnostic Explanations (LIME)

LIME is a technique for explaining black-box models, or models whose inner logic is obscure and difficult to comprehend [47]. LIME adjusts the feature values for a single data sample and monitors its impact on the output. In this method, data around an instance are simulated via random perturbation, and specific selection techniques are employed to assess the significance of attributes. Accordingly, a feature selection process is developed, which selects the features from the new data that best characterize the model result. Finally, a straightforward model is developed, fitted to the newly chosen data, and used to create an explanation for the intended model [48].

**Figure 6:**
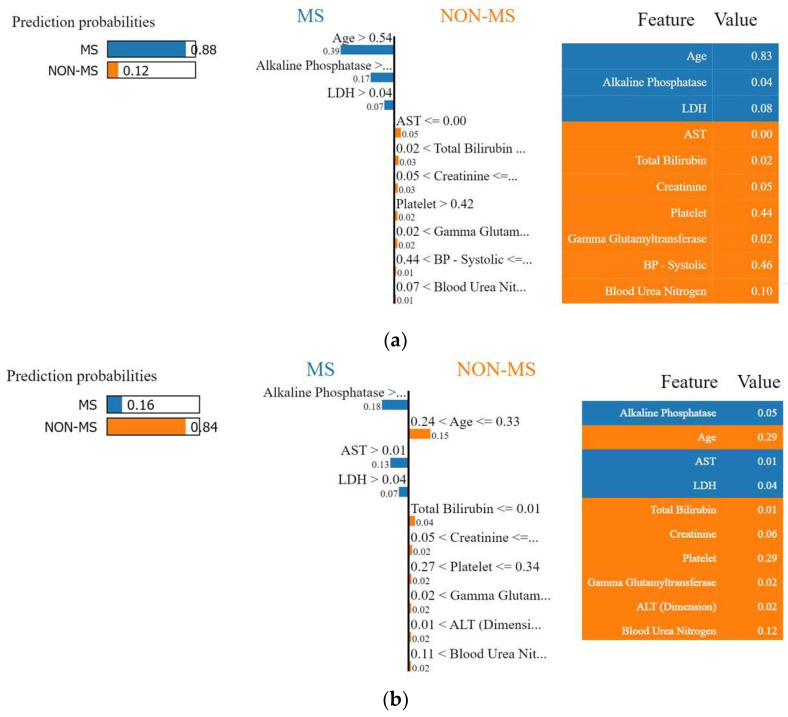
Lime results for the ET model. (**a**) Positive prediction probability, (**b**) negative prediction probability.

The positive probability prediction generated by the ET model, shown in Figure 6a, was 88%. The figure reveals that features including “Age”, “Alkaline Phosphatase”, and “LDH” contributed to the correct classification of the model for pwMS. Contradictorily, Figure 6b explains the negative prediction generated by the ET model, where the negative probability prediction was 84%. The figure reveals that all features except for “Alkaline Phosphatase”, “AST”, and “LDH” have contributed to the correct prediction of non-MS patients.

## 5. Discussion

With the significant advancement of technology in the past couple of decades, emerging technologies, such as ML, have shown a promising revolution in healthcare. The value of ML lies in its ability to interpret the vast amounts of healthcare data generated daily by electronic health records, allowing healthcare providers to improve and speed up care delivery by evaluating a broader range of data [49]. Moreover, it has been proven that deploying ML models in healthcare systems could contribute significantly in terms of automating primary/tertiary healthcare systems and introducing intelligent decision-making techniques, resulting in lower medical testing costs and time and higher average life expectancy [50].

Over the past two decades, MS has become more prevalent, especially in Saudi Arabia, with a prevalence of approximately 40.4 people per 100,000 of the general population and 61.95 per 100,000 of the Saudi national population [5]. Few pwMS are diagnosed in the early stages of the illness, and pwMS in Saudi Arabia may not be provided with optimum delivery care. Therefore, it is suggested that utilizing ML to pre-emptively diagnose MS may contribute to reducing the associated risks. 

Several studies have been conducted to diagnose pwMS using ML techniques, attaining optimistic outcomes. However, very small datasets with few positively confirmed pwMS were used, which may cause the proposed models to be biased to certain patterns [17,20,22,23]. Additionally, most of the studies that achieved significant outcomes relied on MRI images, which are known to have a large sample requirement. Additionally, it is observed that the MRI market in Saudi Arabia reached USD 100.14 million in 2021 and is expected to reach USD 140.77 million by 2027 at a compound annual growth rate of 5.61% [51]. Therefore, acquiring MRI data is an inherently costly and lengthy procedure. The long data-capture times result in limited patient throughput, discomfort, and motion artifacts [52]. Accordingly, it is essential to overcome the limitations of the previous work by developing a timely model for detecting MS. As an alternative to MRI, blood testing can be a less invasive and cost-effective test. Globally, blood sample-handling infrastructures and clinical routines for blood testing have already been well established. Accordingly, it can play an essential role in cutting the costs of diagnosing diseases [53]. 

This study’s main objective was to build the first ML model to diagnose MS using demographical data and clinical biomarkers collected from the Eastern Province of Saudi Arabia. Several ML algorithms have been developed and compared to find the best-performing model. The results indicated that, with only minor variations in their performance metrics, practically all models performed similarly. However, the ET model outperformed the remaining proposed models, attaining an accuracy of 94.74%, a recall of 97.26%, a precision of 94.67%, an F1-score of 95.95%, and an AUC of 0.94 using 11 attributes. The SHAP XAI technique showed that features including “Age”, “BP- Systolic”, “Alkaline Phosphatase”, “Platelet”, and “Creatinine” have the greatest impact on the model’s prediction. 

The patient’s age is ranked as the most important feature in screening MS patients from others, which aligns with the research [10]. The research showed that age is a significant predictor for the diagnosis of MS because aging induces changes in the brain. The second most important feature, BP—systolic, was also shown to have a gradient association with MS [54]. Moreover, platelet was proven to have an association with MS by the study [55]. Their investigation aimed to evaluate the MS patients’ platelet adhesiveness. Both pwMS and the control group had blood samples collected, and the final platelet count percentage showed the degree of platelet stickiness. The results showed that platelet stickiness raised in pwMS compared to the control subjects. Additionally, creatine was also proven to have a significant effect on the prediction of MS, where the study [56] showed that MS patients experienced higher levels of creatinine than the control group, which consisted of healthy subjects having the same age and gender. 

The fact that several studies are in line with the SHAP findings shows the reliability of the proposed model. In addition, as opposed to previous studies in the literature, this study focused on the prediction of MS using clinical data instead of MRI imaging, ensuring that the model is computational and cost-efficient. 

## 6. Conclusions

MS is a chronic inflammatory disease that causes long-term functional impairment and disability. It is usually misdiagnosed as other diseases, such as functional neurologic disorders, migraines, and arterial disease, since the symptoms vary depending on the impacted areas and the damage. Furthermore, there are no specific tests that can identify MS with certainty. Therefore, specialists must use a differential diagnosis that depends on ruling out other conditions that might have a similar set of symptoms. Few people have been diagnosed accurately in the early phases of their disease and receive timely care that contributes to reducing the course of the disease, which proves the importance of early screening and diagnosis in preventing complications that negatively impact patients’ quality of life. Accordingly, this study aimed to propose a clinically applicable and cost-effective ML model to screen MS pre-emptively using clinical laboratory biomarkers. According to the comparison between the results achieved from the implemented models, the ET classifier outperformed other models with an accuracy of 94.74%, a precision of 94.67%, a recall of 97.26%, and an F1-score of 95.95% using 11 features after using the SelectKBest approach. Furthermore, XAI was employed to ensure that medical specialists could easily understand how the algorithms could comprehend or interpret the judgments and the most relevant features of the predictive model. The findings of SHAP indicated that features including “Age”, “BP-Systolic”, and “Alkaline Phosphatase” have significant importance on the model prediction. 

Since the occurrence of MS cases is greater in the central part than in the eastern part of KSA, it is recommended to collect data from different regions that could improve and verify the obtained results for future work. Consequently, the trained algorithm will be evaluated on new patients to confirm its dependability level and improve it to acquire better accuracy. Additionally, the model could be upgraded to diagnose the stages of MS reliably.

## Figures and Tables

**Table 1:** Features’ description.

Feature	Type
Sex	Categorical
Age	Integer
Anion Gap	Integer
ALT (Dimension)	Integer
LDH	Integer
White Blood Cells	Float
Red Blood Cells	Float
Hemoglobin	Float
Hematocrit	Float
Sodium	Integer
Potassium	Float
Chloride	Integer
Carbon Dioxide	Integer
Creatinine	Float
Total Protein	Float
Albumin	Float
Blood Urea Nitrogen	Integer
Total Bilirubin	Float
Direct Bilirubin	Float
Gamma Glutamyl transferase	Integer
MCV	Float
MCH	Float
MCHC	Float
Alkaline Phosphatase	Integer
RDW	Float
MPV	Float
AST	Integer
Lymphocyte—Instrument %	Float
Monocyte—Instrument %	Float
Lymphocyte—Instrument Abso	Float
Monocyte—Instrument Abso	Float
Neutrophil Granulocyte—Instrument %	Float
Neutrophil Granulocyte—Instrument Abso	Float
Platelet	Integer
Eosinophil—Instrument %	Float
Eosinophil—Instrument Abso	Float
Basophil—Instrument %	Float
Basophil—Instrument Abso	Float
BP—Systolic	Integer
Pulse Ox	Integer
Temperature	Float
Pulse	Integer
Respiratory Rate	Integer
BP—Diastolic	Integer
Class	Boolean

**Table 2:** Statistical analysis of numerical features.

Feature	Mean	Standard Deviation	Min	25th Quartile	50th Quartile	75th Quartile	Max	Missing Value Counts
Age	43.28	16.71	13.00	30.00	38.00	55.00	89.00	0
Anion Gap	9.61	2.63	1.00	8.00	10.00	11.00	27.00	20
ALT (Dimension)	33.59	64.25	5.00	16.00	22.00	32.00	1278.00	12
LDH	182.51	131.29	95.00	142.75	164.00	187.00	2523.00	13
White Blood Cells	6.39	2.62	1.30	4.65	6.00	7.60	28.20	2
Red Blood Cells	4.68	0.69	1.91	4.26	4.69	5.13	6.72	2
Hemoglobin	12.80	2.14	5.80	11.50	12.90	14.40	18.50	2
Hematocrit	38.65	5.99	18.30	35.10	38.90	42.90	54.30	2
Sodium	140.00	2.69	125.00	138.00	140.00	141.00	157.00	20
Potassium	4.30	0.43	3.10	4.00	4.30	4.50	6.10	20
Chloride	103.86	2.51	92.00	102.00	104.00	105.00	121.00	20
Carbon Dioxide	26.43	3.14	13.00	25.00	27.00	28.00	44.00	20
Creatinine	0.89	0.97	0.15	0.62	0.72	0.89	12.23	19
Total Protein	7.31	0.64	3.10	7.00	7.30	7.70	8.90	13
Albumin	3.80	0.52	1.70	3.60	3.90	4.10	5.10	13
Blood Urea Nitrogen	13.81	9.37	3.00	9.00	12.00	15.00	83.00	20
Total Bilirubin	0.62	0.98	0.10	0.30	0.40	0.70	16.70	13
Direct Bilirubin	0.21	0.56	0.05	0.09	0.12	0.20	11.10	13
Gamma Glutamyl Transferase	49.85	104.49	4.00	19.00	27.00	43.00	1296.00	13
MCV	83.41	8.81	48.9	79.35	84.50	89.20	133.00	2
MCH	27.48	3.29	15.30	25.80	28.00	29.80	40.90	2
MCHC	33.00	1.39	27.70	32.10	33.10	34.00	36.20	2
Alkaline Phosphatase	85.00	100.13	21.00	58.00	70.00	90.00	1881.00	13
RDW	14.39	2.25	10.40	13.10	13.80	15.00	30.90	2
MPV	8.77	1.02	6.00	8.10	8.70	9.40	12.70	4
AST	34.97	157.53	5.00	15.00	18.00	24.00	2560.00	12
Lymphocyte—Instrument %	33.91	11.95	2.00	26.60	34.30	42.30	69.20	43
Monocyte—Instrument %	8.78	2.54	1.20	7.00	8.40	10.17	19.20	43
Lymphocyte—Instrument Abso	2.06	0.82	0.20	1.50	2.00	2.60	5.40	43
Monocyte—Instrument Abso	0.54	0.20	0.10	0.40	0.50	0.60	1.60	43
Neutrophil Granulocyte—Instrument %	53.87	13.37	17.70	45.30	53.30	60.70	91.90	43
Neutrophil Granulocyte—Instrument Abso	3.69	2.00	0.70	2.40	3.30	4.60	12.30	43
Platelet	254.80	81.72	27.00	204.00	249.00	302.25	679.00	9
Eosinophil—Instrument %	2.67	2.00	0.00	1.30	2.20	3.60	13.70	43
Eosinophil—Instrument Abso	0.16	0.15	0.00	0.10	0.10	0.20	1.00	43
Basophil—Instrument %	0.60	0.42	0.00	0.30	0.50	0.80	2.80	43
Basophil—Instrument Abso	0.03	0.04	0.00	0.00	0.00	0.10	0.20	43
BP—Systolic	126.30	18.01	58.00	115.00	124.00	137.00	191.00	110
Pulse Ox	98.70	2.56	53.00	98.00	99.00	100.00	100.00	123
Temperature	36.76	0.29	35.40	36.60	36.80	36.90	39.30	120
Pulse	83.40	13.00	50.00	75.00	83.00	89.00	132.00	111
Respiratory Rate	19.59	1.29	15.00	18.00	20.00	20.00	28.00	110
BP—Diastolic	76.00	10.92	32.00	69.00	76.00	84.00	107.00	110

**Table 3:** The optimal hyperparameters for each classifier.

Classifier	Hyperparameter	Optimal Hyperparameter
SVM	C	1
	gamma	1
	kernel	rbf
DT	criterion	gini
	max_depth	2
	min_samples_leaf	3
	max_leaf_nodes	19
LR	penalty	l2
	class_weight	dict
	C	1
RF	max_depth	6
	n_estimators	150
	criterion	entropy
	max_leaf_nodes	None
	min_samples_leaf	3
XGBoost	n_estimators	80
	learning_rate	0.1
	booster	gbtree
	gamma	0.3
AdaBoost	n_estimators	100
	learning_rate	0.2
	algorithm	SAMME.R
ET	n_estimators	450
	max_depth	12
	max_leaf_nodes	None
	min_samples_leaf	1

**Table 4:** The results of the proposed models using the ideal hyperparameters.

Classifier	Training Accuracy	Testing Accuracy	Precision	Recall	F1-Score
SVM	89.01%	92.98%	94.52%	94.52%	94.52%
DT	88.13%	92.11%	93.24%	94.52%	93.88%
LR	87.69%	92.98%	94.52%	94.52%	94.52%
RF	92.53%	92.98%	93.33%	95.89%	94.59%
XGBoost	99.56%	93.86%	94.59%	95.89%	95.24%
AdaBoost	93.24%	92.11%	93.24%	94.52%	93.88%
**ET**	95.82%	94.74%	94.67%	97.26%	95.95%

## Data Availability

The dataset is available upon request from the corresponding author.

## Supplementary Materials

The following supporting information can be downloaded at: www.mdpi.com/article/10.3390/ijerph20054261/s1, Supplementary file.
