# Supplementary material for: Comprehensible Machine-Learning-Based Models for the Pre-Emptive Diagnosis of Multiple Sclerosis Using Clinical Data: A Retrospective Study in the Eastern Province of Saudi Arabia
Source: Int J Environ Res Public Health. 2023 Feb 27;20(5):4261. doi: 10.3390/ijerph20054261 (PMC10002108; doi:10.3390/ijerph20054261)
Supplement: Supplementary file 1 [file ijerph-20-04261-s001.zip › ijerph-2213754-supplementary.pdf]

The hyperparameters tuned for the SVM classifier included the C value, gamma, and kernel, as follows:

- C values: {1, 2, 3, 4, 10, 12, 14, 18, 19, 20, 21, 22, 30}.
- Gamma values: {1, 0.1, 0.01, 0.001, 0.0001, "auto", "scale"}.
- Kernel values: {"rbf", "linear", "poly", "sigmoid"}.

The hyperparameters tuned for the DT classifier included the criterion, max_depth, min_samples_leaf, and max_leaf_nodes, as follows:

- Criterion values: {"gini", "entropy", "log_loss"}.
- Max_depth values: {1, 2, 3, 4, 5, 6, 7, 8, 9, 10, 11, 12, None}.
- Min_samples_leaf ranged from 1 to 29.
- Max_leaf_nodes values: {1, 2, 3, 4, 5, 6, 8, 9, 10, 11, 12, 20, 22, 24, 25, 28, 30}.

The hyperparameters tuned for the LR classifier included the penalty, class_weight, and C value.

- Penalty values: {"l1", "l2", "elasticnet", "none"}.
- Class_weight values: {"dict", "balanced", None}.
- C values: {1, 0.5, 0.01, 0.001, 2, 3, 4, 5, 6, 7, 8, 9, 10}.

The hyperparameters tuned for the RF classifier included the max_depth, n_estimators, criterion, max_leaf_nodes, and min_samples_leaf.

- Max_depth values: {2, 4, 6, 8, 10, 12, 14, 16, 20, 25, 30, None}.
- N_estimators ranged from 100 to 350, with 50 steps in each iteration.
- Criterion values: {"gini", "entropy", "log_loss"}.
- Max_leaf_nodes values: {2, 4, 6, 8, 10, 12, 14, 16, None}.
- Min_samples_leaf values ranged from 1 to 10.

The hyperparameters tuned for the XGBoost classifier included the n_estimators, learning_rate, booster, and gamma.

- N_estimators ranged from 50 to 290, with 10 steps in each iteration.
- Learning_rate values: {0.01, 0.05, 0.1, 0.2, 0.4, 0.5, 1}.
- Booster values: {"gbtree", "gblinear", "dart"}.
- Gamma values: {0, 0.5, 1, 0.3, 0.4, 0.5, 1, 2, 3}.

The hyperparameters tuned for the Adaboost classifier included the n_estimators, learning_rate, and algorithm.

- N_estimators ranged from 50 to 390, with 10 steps in each iteration.
- Learning_rate values: {0.0001, 0.001, 0.01, 0.1, 0.2, 0.3, 0.5, 1, 2, 3}.
- Algorithm values: {"SAMME", "SAMME.R"}.

The hyperparameters tuned for the ET classifier included the n_estimators, max_depth, max_leaf_nodes, and min_samples_leaf.

- N_estimators values ranged from 50 to 550, with 50 steps in each iteration.
- Max_depth values: {1, 2, 3, 4, 5, 6, 7, 8, 9, 10, 12, 13, 14, 16}.
- Max_leaf_nodes values: {2, 4, 6, 8, 10, 12, 14, 16, None}.
- Min_samples_leaf values ranged from 1 to 10.
